# Supplementary material for: The riboflavin biosynthetic pathway as a novel target for antifungal drugs against Candida species
Source: mBio. 2024 Oct 15;15(11):e02502-24. doi: 10.1128/mbio.02502-24 (PMC11559065; doi:10.1128/mbio.02502-24)
Supplement: Supplemental material — Supplemental figures and tables. [file mbio.02502-24-s0001.docx]

Supplementary Tables:

Supplementary Table1:

| **Strain name** | **Genotype** | **Reference** | **Identifier** |
| --- | --- | --- | --- |
| *Candida albicans*  SC5314 | Wild type strain, clinical *Candida albicans* isolate | ATCC (1) | Cat#MYA-2876 |
| *Carib1∆/∆* | SC5314 with *Carib1∆/∆* | (2) | JN19, JN20, JN21 |
| *Carib2∆/∆* | SC5314 with *Carib2∆/∆* | This study | JN22, JN23, JN24 |
| *Carib3∆/∆* | SC5314 with *Carib3∆/∆* | This study | JN25, JN26, JN27 |
| *Carib4∆/∆* | SC5314 with *Carib4∆/∆* | This study | JN28, JN29, JN30 |
| *Carib5∆/∆* | SC5314 with *Carib5∆/∆* | This study | JN31, JN32, JN33 |
| *Carib7∆/∆* | SC5314 with *Carib7∆/∆* | This study | JN34, JN35, JN36 |
| *Carib1∆/∆ + RIB1* | *Carib1*∆/∆, with CIp10-*CaRIB1-NAT1* integrated | This study | JN37, JN38, JN39 |
| *Carib2∆/∆ + RIB2* | *Carib1*∆/∆, with CIp10-*CaRIB2-NAT1* integrated | This study | JN40, JN41, JN42 |
| *Carib3∆/∆ + RIB3* | *Carib1*∆/∆, with CIp10-*CaRIB3-NAT1* integrated | This study | JN43, JN44, JN45 |
| *Carib4∆/∆ + RIB4* | *Carib1*∆/∆, with CIp10-*CaRIB4-NAT1* integrated | This study | JN46, JN47, JN48 |
| *Carib5∆/∆ + RIB5* | *Carib1*∆/∆, with CIp10-*CaRIB5-NAT1* integrated | This study | JN49, JN50, JN51 |
| *Carib7∆/∆ + RIB7* | *Carib1*∆/∆, with CIp10-*CaRIB7-NAT1* integrated | This study | JN52, JN53, JN54 |
| *CaRIB1/rib1Δ* | SC5314 with *RIB1/rib1Δ* | This study | JN1, JN2, JN3 |
| *CaRIB2/rib2Δ* | SC5314 with *RIB2/rib2Δ* | This study | JN4, JN5, JN6 |
| *CaRIB3/rib3Δ* | SC5314 with *RIB3/rib3Δ* | This study | JN7, JN8, JN9 |
| *CaRIB4/rib4Δ* | SC5314 with *RIB4/rib4Δ* | This study | JN10, JN11, JN12 |
| *CaRIB5/rib5Δ* | SC5314 with *RIB5/rib5Δ* | This study | JN13, JN14, JN15 |
| *CaRIB7/rib7Δ* | SC5314 with *RIB7/rib7Δ* | This study | JN16, JN17, JN18 |
| *Caorf19.6263∆/∆* | SC5314 with *orf19.6263∆/∆* | This study | JN55, JN56, JN57 |
| *Caorf19.5720∆/∆* | SC5314 with *orf19.5720∆/∆* | This study | JN58, JN59, JN60 |
| *Caorf19.1584∆/∆* | SC5314 with *orf19.1584∆/∆* | This study | JN61, JN62, JN63 |
| *Caorf19.2751∆/∆* | SC5314 with *orf19.2751∆/∆* | This study | JN64, JN65, JN66 |
| *Caorf19.4337∆/∆* | SC5314 with *orf19.4337∆/∆* | This study | JN67, JN68, JN69 |
| *Carib1∆/∆ orf19.6263∆/∆* | *Carib1∆/∆* with *orf19.6263∆/∆* | This study | JN70, JN71, JN72 |
| *Carib1∆/∆ orf19.5720∆/∆* | *Carib1∆/∆* with with *orf19.5720∆/∆* | This study | JN73, JN74, JN75 |
| *Carib1∆/∆ orf19.1584∆/∆* | *Carib1∆/∆* with *orf19.1584∆/∆* | This study | JN76, JN77, JN78 |
| *Carib1∆/∆ orf19.2751∆/∆* | *Carib1∆/∆* with *orf19.2751∆/∆* | This study | JN79, JN80, JN81 |
| *Carib1∆/∆ orf19.4337∆/∆* | *Carib1∆/∆* with *orf19.4337∆/∆* | This study | JN82, JN83, JN84 |
| *Carib1∆/∆ orf19.6263∆/∆ orf19.5720∆/∆ orf19.2751∆/∆ orf19.1584∆/∆* | *Carib1∆/∆* with *orf19.6263∆/∆, orf19.5720∆/∆, orf19.2751∆/∆, orf19.1584∆/∆* | This study | JN85, JN86, JN87 |
| *Carib1∆/∆ orf19.6263∆/∆ orf19.5720∆/∆ orf19.2751∆/∆ orf19.1584∆/∆*  *Orf19.4337∆/∆* | *Carib1∆/∆* with *orf19.6263∆/∆, orf19.5720∆/∆, orf19.2751∆/∆, orf19.1584∆/∆, orf19.4337∆/∆* | This study | JN88, JN89, JN90 |
| *Candida glabrata* ATCC2001 (CBS138) | wild-type strain | (3) | 4309 |
| *Cgrib1∆* | ATCC2001 *rib1∆::FRT* | This study | JN91, JN92, JN93 |
| *Cgrib2∆* | ATCC2001 *rib2∆::FRT* | This study | JN94, JN95, JN96 |
| *Cgrib3∆* | ATCC2001 *rib3∆::FRT* | This study | JN97, JN98, JN99 |
| *Cgrib4∆* | ATCC2001 *rib4∆::FRT* | This study | JN100, JN101, JN102 |
| *Cgrib5∆* | ATCC2001 *rib5∆::FRT* | This study | JN103, JN104, JN105 |
| *Cgrib7∆* | ATCC2001 *rib7∆::FRT* | This study | JN106, JN107, JN108 |
| *Cgrib1∆ + RIB1* | *Cgrib1*∆/∆, with pYC44-*CgRIB1* integrated | This study | JN140, JN141, JN142 |
| *S. cerevisiae*  S288C | Wild-type | (4) | JT1 |
| *Scrib1∆* | S288C with rib1∆::HPH | This study | JN109, JN110, JN111 |
| *Scrib2∆* | S288C with *rib2∆:*:HPH | This study | JN112, JN123, JN124 |
| *Scrib3∆* | S288C with *rib3∆:*:HPH | This study | JN125, JN126, JN127 |
| *Scrib4∆* | S288C with *rib4∆:*:HPH | This study | JN128, JN129, JN130 |
| *Scrib5∆* | S288C with *rib5∆:*:HPH | This study | JN131, JN132, JN133 |
| *Scrib7∆* | S288C with *rib7∆:*:HPH | This study | JN134, JN135, JN136 |
| *Scrib1∆ mch5∆* | *Scrib1∆* with *mch5∆:*:HPH | This study | JN137, JN138, JN139 |

Supplementary Table 2: plasmids used in this study.

| **Plasmid name** | **Backbone** | **Insert of interest** | **Marker** | **Reference** |
| --- | --- | --- | --- | --- |
| **Hernday Lab *C. albicans* CRISPR system** | | | | |
| pADH110 | pUC19 | pSNR52 promoter fragment | *AmpR* | **(5)** |
| pADH147 | pUC19 | "empty" gRNA construct | *AmpR* | **(5)** |
| pADH99 | pUC19 | *CAS9* | *AmpR* | **(5)** |
| **SAT1-flipper** | | | | |
| pSFS2 | / | */* | Amp^R^ | (6) |
| pSFS2-*RIB1* | pSFS2 | *RIB1* | Amp^R^ | This study |
| pSFS2-*RIB2* | pSFS2 | *RIB2* | Amp^R^ | This study |
| pSFS2-*RIB3* | pSFS2 | *RIB3* | Amp^R^ | This study |
| pSFS2-*RIB4* | pSFS2 | *RIB4* | Amp^R^ | This study |
| pSFS2-*RIB5* | pSFS2 | *RIB5* | Amp^R^ | This study |
| pSFS2-*RIB7* | pSFS2 | *RIB7* | Amp^R^ | This study |
| ***C. albicans* reintgrant strains** | | | | |
| Cip10-*NAT1* | / | */* | *AmpR* | (7) |
| CIp10-*RIB1-NAT1* | CIp10-*NAT1* | *CaRIB1* | *AmpR* | This study |
| CIp10-*RIB2-NAT1* | CIp10-*NAT1* | *CaRIB2* | *AmpR* | This study |
| CIp10-*RIB3-NAT1* | CIp10-*NAT1* | *CaRIB3* | *AmpR* | This study |
| CIp10-*RIB4-NAT1* | CIp10-*NAT1* | *CaRIB4* | *AmpR* | This study |
| CIp10-*RIB5-NAT1* | CIp10-*NAT1* | *CaRIB5* | *AmpR* | This study |
| CIp10-*RIB7-NAT1* | CIp10-*NAT1* | *CaRIB7* | *AmpR* | This study |
| ***C. glabrata* gene deletions** | | | | |
| pYC44 | / | */* | AMP^R^ NAT^R^ *URA3*+ | (8) |
| pYC44-*RIB1-*HR | pYC44 | *RIB1* up- and downstream homologous regions | AMP^R^ NAT^R^ *URA3*+ | This study |
| pYC44-*RIB2-*HR | pYC44 | *RIB2* up- and downstream homologous regions | AMP^R^ NAT^R^ *URA3*+ | This study |
| pYC44-*RIB3-*HR | pYC44 | *RIB3* up- and downstream homologous regions | AMP^R^ NAT^R^ *URA3*+ | This study |
| pYC44-*RIB4-*HR | pYC44 | *RIB4* up- and downstream homologous regions | AMP^R^ NAT^R^ *URA3*+ | This study |
| pYC44-*RIB5-*HR | pYC44 | *RIB5* up- and downstream homologous regions | AMP^R^ NAT^R^ *URA3*+ | This study |
| pYC44-*RIB7-*HR | pYC44 | *RIB7* up- and downstream homologous regions | AMP^R^ NAT^R^ *URA3*+ | This study |
| pLS10 | / | Expresses *FLP1*, HPH marker | AMP^R^, HPH^R^ | (9) |
| pYC44-*RIB1-*HR + *cgRIB1* | pYC44 | *RIB1* up- and downstream homologous regions; with *cgRIB1* gene integrated instead of NAT: used for reintegration of *RIB1* in original locus | AMP^R^ *URA3*+ | This study |
| ***S. cerevisiae* gene deletions** | | | | |
| pTOPO-A1-G2-B-HPH-P-G2-A2(p79) | pTOPO2.1 | knock-out of genes with possibility for CrispR /Cas mediated scarless loop-out | AMP^R^, KanR, HPH^R^ | Lab of molecular cell biology; Ku Leuven(10) |
| pTOPO-A1-G2-B-KanMX-P-G2-A2(p76) | pTOPO2.1 | knock-out of genes with possibility for CrispR /Cas mediated scarless loop-out | AMP^R^, Kan^R^, kan^MX^ | Lab of molecular cell biology; Ku Leuven(10) |

HR: homologous regions; AMP^R^: ampicillin resistance gene; NAT^R^: nourseothricin resistance gene; URA3+: URA3 selection marker; HPH: hygromycin B phosphotransferase; KanR: kanamycin; kanMX: geneticin G418

Supplementary Table 3: primers used in this study

| **Primer name** | **Sequence 5’ to 3’** |
| --- | --- |
| **Generation of *C. albicans* homozygous deletion strains** | |
| AHO1096 | GACGGCACGGCCACGCGTTTAAACCGCC |
| AHO1098 | CAAATTAAAAATAGTTTACGCAAG |
| AHO1097 | CCCGCCAGGCGCTGGGGTTTAAACACCG |
| AHO1236 | TAAAGCTGCCACAAGAGGTATTTC |
| AHO1237 | AGGTGATGCTGAAGCTATTGAAG |
| *RIB2* gRNA oligo | cgtaaactatttttaatttgGGGTTCGACCAGTTAATGGGgttttagagctagaaatagc |
| *RIB3* gRNA oligo | cgtaaactatttttaatttgCCTGGTTTAATGAAATCTTGgttttagagctagaaatag |
| *RIB4* gRNA oligo | cgtaaactatttttaatttgCAGCACCCCAATCTTCACCAgttttagagctagaaatag |
| *RIB5* gRNA oligo | cgtaaactatttttaatttgAATCTCCTAGATTGGTTCTGgttttagagctagaaatagc |
| *RIB7* gRNA oligo | cgtaaactatttttaatttgGTATCAGTACCAGTACCAGCgttttagagctagaaatagc |
| orf19.6263 gRNA oligo | cgtaaactatttttaatttgGCCAATGCCAAGATAAACTGgttttagagctagaaatag |
| orf19.5720 gRNA oligo | cgtaaactatttttaatttgCCATCTTCCTAAAATACCCGgttttagagctagaaatagc |
| orf19.1584 gRNA oligo | cgtaaactatttttaatttgAGCTCTGCTATCGTATCGAGgttttagagctagaaatagc |
| orf19.2751 gRNA oligo | cgtaaactatttttaatttgAAATGGTCCAAATACAAACGgttttagagctagaaatagc |
| Orf19.4337 gRNA oligo | cgtaaactatttttaatttgAACAAGACAAAAGAATGACAgttttagagctagaaatagc |
| *RIB2* donor DNA FW | TCTTGTTGGGAAAAAGGAAAAACTTGATACTGGATTGTCCTTTGCTGAGATGTCCTTATATTTTATTCATCACTAGATACATCTAATAT |
| *RIB2* donor DNA RV | TATATTAGATGTATCTAGTGATGAATAAAATATAAGGACATCTCAGCAAAGGACAATCCAGTATCAAGTTTTTCCTTTTTCCCAACAAG |
| *RIB3* donor DNA FW | ATATATCCTTTCAAATAACCAACTCCCTTTCTTTATATATATATTTTCAACGCACAAACGGAAGAAACAAAATAGAACATATACATTTA |
| *RIB3* donor DNA RV | TTAAATGTATATGTTCTATTTTGTTTCTTCCGTTTGTGCGTTGAAAATATATATATAAAGAAAGGGAGTTGGTTATTTGAAAGGATATA |
| *RIB4* donor DNA FW | ATTTGCCGCCTGTATATAAGCATGTGAACACCAGATAATTATAATGTATATATATAGAGTTTTGCGCCTGAATAAATGATCTTATATGA |
| *RIB4* donor DNA RV | TTCATATAAGATCATTTATTCAGGCGCAAAACTCTATATATATACATTATAATTATCTGGTGTTCACATGCTTATATACAGGCGGCAAA |
| *RIB5* donor DNA FW | CTCTTTCTCACCCCCTCAAGCAAACTACAATTATTTCTATCAACTTGAATTGTATAGCAAAAGTAATGTACAAATTCTCTTTTCAGAAC |
| *RIB5* donor DNA RV | CGTTCTGAAAAGAGAATTTGTACATTACTTTTGCTATACAATTCAAGTTGATAGAAATAATTGTAGTTTGCTTGAGGGGGTGAGAAAGA |
| *RIB7* donor DNA FW | AGCCAATCGACCCACTTAGTGTCAATTATTTACCTAAATTAAAACTTTACTTTACTTTACTTTACTTTACTTTACTTTACTTTATTTAA |
| orf19.6263 donor DNA FW | CTATTCTATTTCTACCACTTCAAATGACATAAGATTACCCTCATTTATAATACATACATGCATACTATTGGCTATCGTCTCTTTTTAGC |
| orf19.6263 donor DNA RV | TGCTAAAAAGAGACGATAGCCAATAGTATGCATGTATGTATTATAAATGAGGGTAATCTTATGTCATTTGAAGTGGTAGAAATAGAATA |
| orf19.5720 donor DNA FW | CAATTTTCCTAGATATACCTGGTTTTATTACAAATACCCAACTCAGTACTGACTTGTTGTGTCTATATTGCAATCATTAGTAAATTCAT |
| orf19.5720 donor DNA RV | CATGAATTTACTAATGATTGCAATATAGACACAACAAGTCAGTACTGAGTTGGGTATTTGTAATAAAACCAGGTATATCTAGGAAAATT |
| orf19.1584 donor DNA FW | ATATGTTGAAATGGCTAGATAAAGTATTCTATTCTTCAGAGACAAATAGGTTATCGAGTGTTTGCTGCAATCTACAGTGTAAATGTATT |
| orf19.1584 donor DNA RV | AAATACATTTACACTGTAGATTGCAGCAAACACTCGATAACCTATTTGTCTCTGAAGAATAGAATACTTTATCTAGCCATTTCAACATA |
| orf19.2751 donor DNA FW | TATCATTCCCTTTCTTCCAAGTTGAATTAATGATATAGTTATAATTACAATTCATGACGAAAACAACAACAATAAAAGTTAATAAATAT |
| orf19.2751 donor DNA RV | TATATTTATTAACTTTTATTGTTGTTGTTTTCGTCATGAATTGTAATTATAACTATATCATTAATTCAACTTGGAAGAAAGGGAATGAT |
| orf19.4337 dDNA FW | TATAGTCTTTTTTTAAAAAAAACAAAGTATAAACACATCTCAAAATCAGTGTATAGAAAAATAGGTTAGCTTATAAGATCGAGAGACAC |
| orf19.4337 dDNA RV | GTGTCTCTCGATCTTATAAGCTAACCTATTTTTCTATACACTGATTTTGAGATGTGTTTATACTTTGTTTTTTTTAAAAAAAGACTATA |
| **Generation of *C. albicans* heterozygous deletion strains** | |
| *RIB1* upstream FW | AAGGGAACAAAAGCTGGGTACCGGGCCCGCACGAGTGGTCTATAAATG |
| *RIB1* upstream RV | TATTCTCTAGAAAGTATAGGAACTTCCTCGAGGGGTTGTGGTAGATTCTGTA |
| *RIB1* downstream FW | GAACTTCAGATCCACTAGTTCTAGAGCGGCCGCATTGTTATGGAGTTGATGAG |
| *RIB1* downstream RV | CGACTCACTATAGGGCGAATTGGAGCTCCTTCACTATCAGATGATGAG |
| *RIB2* upstream FW | AAGGGAACAAAAGCTGGGTACCGGGCCCGCTATACAAGCTACAAGGAC |
| *RIB2* upstream RV | TATTCTCTAGAAAGTATAGGAACTTCCTCGAGAGGACAATCCAGTATCAAGT |
| *RIB2* downstream FW | GAACTTCAGATCCACTAGTTCTAGAGCGGCCGCTGAGATGTCCTTATATTTTATTC |
| *RIB2* downstream RV | CGACTCACTATAGGGCGAATTGGAGCTCCTCCTGGCAATGAACTAAAT |
| *RIB3* upstream FW | AAGGGAACAAAAGCTGGGTACCGGGCCCGGTTATGATTATGCCAATTC |
| *RIB3* upstream RV | TATTCTCTAGAAAGTATAGGAACTTCCTCGAGGAAAGGGAGTTGGTTATTTG |
| *RIB3* downstream FW | GAACTTCAGATCCACTAGTTCTAGAGCGGCCGCAACGCACAAACGGAAGAAAC |
| *RIB3* downstream RV | CGACTCACTATAGGGCGAATTGGAGCTCGCCGGTGCTATAGGTTATGA |
| *RIB4* upstream FW | AAGGGAACAAAAGCTGGGTACCGGGCCCCGGTCGTATTCAATAACTGG |
| *RIB4* upstream RV | TATTCTCTAGAAAGTATAGGAACTTCCTCGAGCATGCTTATATACAGGCGGC |
| *RIB4* downstream FW | GAACTTCAGATCCACTAGTTCTAGAGCGGCCGCGAGTTTTGCGCCTGAATAAA |
| *RIB4* downstream RV | CGACTCACTATAGGGCGAATTGGAGCTCAGATTACGCACCATAACTCT |
| *RIB5* upstream FW | AAGGGAACAAAAGCTGGGTACCGGGCCCCTAGTAACTTCGTGTTCTGC |
| *RIB5* upstream RV | TATTCTCTAGAAAGTATAGGAACTTCCTCGAGATTGTAGTTTGCTTGAGGGG |
| *RIB5* downstream FW | GAACTTCAGATCCACTAGTTCTAGAGCGGCCGCCTCTTTTCAGAACGCTCCGC |
| *RIB5* downstream RV | CGACTCACTATAGGGCGAATTGGAGCTCCCATGAGCACCATAAGGGAG |
| *RIB7* upstream FW | AAGGGAACAAAAGCTGGGTACCGGGCCCGTGGTGATAATTCAAGTGAA |
| *RIB7* upstream RV | TATTCTCTAGAAAGTATAGGAACTTCCTCGAGTAATTGACACTAAGTGGGTC |
| *RIB7* downstream FW | GAACTTCAGATCCACTAGTTCTAGAGCGGCCGCTTTTCTTGAAGAATTTTCTC |
| *RIB7* downstream RV | CGACTCACTATAGGGCGAATTGGAGCTCCATTAGAAGAATTTCGATCC |
| **Primers used to verify mutants (primers outside the gene)** | |
| *RIB2* FW | GAAGTGGAGGATAAAGAAGG |
| *RIB2* RV | GTTCCAGATTTTAGCCCTGA |
| *RIB3* FW | CTGGTGATGGAAATAGTGAG |
| *RIB3* RV | GGTATTGTGGTTTTAGTGCC |
| *RIB4* FW | GTTCAGATAGGGGTGAAAGA |
| *RIB4* RV | CATCACTACAGATCACCAAC |
| *RIB5* FW | GTCACATTCAATCACAGCCG |
| *RIB5* RV | TTGCAATAAGGGTGCCGTCA |
| *RIB7* FW | GGGGTTTGTTTGTTAGTTGG |
| *RIB7* RV | CAACGAGAAGATCCTGTAGC |
| orf19.6263 FW | GTCCCGTATTGGCATCCGGA |
| orf19.6263 RV | CCGAGTTTGCTGCGGAATTG |
| orf19.5720 FW | CGGACAATAAACTCCGATGA |
| orf19.5720 RV | GACACGGTCTCGTATCCAAT |
| orf19.1584 FW | GGATGCGTGTATCAAATGCA |
| orf19.1584 RV | AATCACATATTCCATTCCCC |
| orf19.2751 FW | TGTATCCTCCAACGGATGGG |
| orf19.2751 RV | CCTCCTTCTCTTAGTCCTGG |
| orf19.4337 FW | CCACTACTACCTTTATCTAAGC |
| orf19.4337 RV | TGGGGGAAACTGTCATTATTAC |
| **Primers used to verify mutants (primers inside the gene)** | |
| *RIB1* FW | TACTGTAAGTGCTAGCAGCC |
| *RIB1* RV | CCCAAATCAACCAAGATAGCC |
| *RIB2* FW | GTGGCTGGGTTTCCCCTTTG |
| *RIB2* RV | GGTACTTCTCTTTCCCCACCA |
| *RIB3* FW | TATGGACGATGAAGACCGTG |
| *RIB3* RV | CAAATCACCCCAGCAGGTT |
| *RIB4* FW | GACCAAAACTGATAATCAGG |
| *RIB4* RV | TGTGCATTTTGCCTTCAATC |
| *RIB5* FW | GGTCTTGTTGAAACCATTGG |
| *RIB5* RV | ATGAAGCCCCATCAATAGCA |
| *RIB7* FW | GTCATTAATCCCATTACCTG |
| *RIB7* RV | ACAGTAACACCATTACAACC |
| **Generation of *C. albicans* reintegrant strains** | |
| *CaRIB1*_Fw_NheI | CATTCAARAATGCTGCAGGCTAGCATGACATCGATAAAACATCCAA |
| *CaRIB1*_Rev_AatII | CGAGGTCGACGGTATCGATGACGTCTTAGATTTTGATGGGTTTCTCTA |
| *CaRIB2*_Fw_NheI | CATTCAAAATGCTGCAGGCTAGCATGTCAATATACAAGAGTGGCT |
| *CaRIB2* _Rev_AatII | CGAGGTCGACGGTATCGATGACGTCTTATTCTTGAATATGTTCATGACC |
| *CaRIB3*_Fw_NheI | CATTCAAAATGCTGCAGGCTAGCATGATCAGTATGACTAACATCTTTA |
| *CaRIB3*_ Rev_AatII | CGAGGTCGACGGTATCGATGACGTCTTATTTAGAAATATATTCAACTAATTG |
| *CaRIB4*_Fw_NheI | CATTCAAAATGCTGCAGGCTAGCATGACCAAAACTGATAATCAGG |
| *CaRIB4*_Rev_AatII | CGAGGTCGACGGTATCGATGACGTCTTAATTGAATTTAGTGGCCATTT |
| *CaRIB5*_Fw_NheI | CATTCAAAATGCTGCAGGCTAGCATGTTTACTGGTCTTGTTGAAAC |
| *CaRIB5*_Rev_AatII | CGAGGTCGACGGTATCGATGACGTCTTACTTGATATATTCTTTAACTTTCT |
| *CaRIB7*_Fw_NheI | CATTCAAAATGCTGCAGGCTAGCATGTCATTAATCCCATTACCTG |
| *CaRIB7* _Rev_AatII | CGAGGTCGACGGTATCGATGACGTCCTATTGATGATAATCAATTCTAG |
| ***C. albicans* qRT-PCR: gene expression** | |
| *RIB1* FW | GTGCCGATACAGTGGAAGC |
| *RIB1* RV | CCCAAATCAACCAAGATAGCC |
| orf19.6263 FW | GGTGCTCTTTATGCATTTGC |
| orf19.6263 RV | ATAATAGCAGCACTCACCGG |
| orf19.5720 FW | GGTGTCGGTGGTTATTGTTC |
| orf19.5720 RV | TCCTTGGGTGTACCATCCTT |
| orf19.1584 FW | GTATATCGTCTTCTGCCGTG |
| orf19.1584 RV | ATGTAGCAAGATACATCCCC |
| orf19.2751 FW | GGGTTGGGTCGTTTCAATAC |
| orf19.2751 RV | CCACTAGCAATTGCTCCAGC |
| orf19,4337 FW | GTGGATCACCTGACGACTTATC |
| orf19,4337 RV | CCATCCCACCATCTGGTTTAT |
| **Primers to test copy number variation** | |
| *CaACT1*pr_Fw | GGCTATGCCAATCAAAAAGG |
| *CaACT1*pr_Rev | CCCCTTGGCCATAGGATATT |
| *CaACT1*_Ref_Fw | TGTTGGTGATGAAGCCCAATC |
| *CaACT1*_Ref_Rev | CATATCGTCCCAGTTGGAAACAA |
| *Ca18S*_Ref_Fw | GATGCCCTTAGACGTTCTGG |
| *Ca18S*_Ref_Rev | CACGACGGAGTTTCACAAGA |
| *CaTEF1*_Ref_Fw | CCACTGAAGTCAAGTCCGTTGA |
| *CaTEF1*_Ref_Rev | CACCTTCAGCCAATTGTTCGT |
| **Generation of *C. glabrata* deletion strains** | |
| *RIB1*_FW_downstream | AGAGAATAGGAACTTCGTCCTACAGTAAAGATCTTTACACAG |
| *RIB1*_RV_downstream | AGCTGGTACCGGGCCCCCCCTCCGCCGGACTCTAACACAGATAAAT |
| *RIB2*_FW_downstream | AGAGAATAGGAACTTCGTCCAAATAGATCAACAAAGTATAGA |
| *RIB2*_RV_ downstream | AGCTGGTACCGGGCCCCCCCTCCGCCCAATTGAGTAAAGATCAAGA |
| *RIB3*_FW_ downstream | AGAGAATAGGAACTTCGTCCGGGTGTTGTTTCTCCGTAAC |
| *RIB3*_RV_ downstream | AGCTGGTACCGGGCCCCCCCTCCGCCCAGGTTTGAAGATACCTCAG |
| *RIB4*_FW_ downstream | AGAGAATAGGAACTTCGTCCATTGTTACCCAACTAACAATG |
| *RIB4*_RV_ downstream | AGCTGGTACCGGGCCCCCCCTCCGCCAACCTGTTCTTCCCTCATTT |
| *RIB5*_FW_ downstream | AGAGAATAGGAACTTCGTCCTTACTTCTCTTATATATGCAGTTTC |
| *RIB5*_RV_ downstream | AGCTGGTACCGGGCCCCCCCTCCGCCGCGATCTGTTAATATTAGTCG |
| *RIB7*_FW_ downstream | AGAGAATAGGAACTTCGTCCTGAATTTCGTTTACCCTGAC |
| *RIB7*_RV_ downstream | AGCTGGTACCGGGCCCCCCCTCCGCCGATTTGCTGTTGTGCCAATC |
| *RIB1*_FW_upstream | CGGCCGCTCTAGAACTAGTGGGCGGACTGTATAACTCACTCTATAC |
| *RIB1*_RV_ upstream | TAGAAAGTATAGGAACTTCGTTGTTGTATTGAATTTGAAT |
| *RIB2*_FW_ upstream | CGGCCGCTCTAGAACTAGTGGGCGGAGGCCATACAAGTGCCTGATC |
| *RIB2*_RV_ upstream | TAGAAAGTATAGGAACTTCGAGTTTCACCAATTAACCTTGC |
| *RIB3*_FW_ upstream | CGGCCGCTCTAGAACTAGTGGGCGGAGGTAATATTACCATACTTGCAGG |
| *RIB3*_RV_ upstream | TAGAAAGTATAGGAACTTCGATTGTAGTAGTTGTAATTGTGC |
| *RIB4*_FW_ upstream | CGGCCGCTCTAGAACTAGTGGGCGGACTAGTAGAATAGTCAGGATATG |
| *RIB4*_RV_ upstream | TAGAAAGTATAGGAACTTCGTGTATAATTATGTATGTTTG |
| *RIB5*_FW_ upstream | CGGCCGCTCTAGAACTAGTGGGCGGAGATATTCTTGTCCTCCTTTC |
| *RIB5*_RV_ upstream | TAGAAAGTATAGGAACTTCGTTTGTTGATCTATTTGTAAGTATGTCCC |
| *RIB7*_FW_ upstream | CGGCCGCTCTAGAACTAGTGGGCGGAGGCAAACAGAGTTAAGTTAT |
| *RIB7*_RV_ upstream | TAGAAAGTATAGGAACTTCGAATTGAATTTGTAGTTGTCTTATC |
| *RIB1*_FW_check | GCTCAATCTTGGTTGTATAC |
| *RIB1*_RV_check | CAAGGTAGGCAATTCATCAG |
| *RIB2*_FW_check | CCATCATGGTGTGACAGAAT |
| *RIB2*_RV_check | GTATATACCGAGGATGTGGC |
| *RIB3*_FW_check | CATGTGATTGATGCGTATTC |
| *RIB3*_RV_check | AGAAGTGGTGAGTTGCTATC |
| *RIB4*_FW_check | TAGTGTGCAACTGAACAATG |
| *RIB4*_RV_check | GAGCAAGAACCCTGAATTAG |
| *RIB5*_FW_check | CTTTATCCGTTATTGCAGCA |
| *RIB5*_RV_check | ACTCTTACCTGTGTTCTAAC |
| *RIB7*_FW_check | AACTATTATACCCAATCCCG |
| *RIB7*_RV_check | CAAGGTCTCTAGTATGTTCA |
| *RIB1*_FW_deletion check | CCAGATATCTTTCTGCACTTG |
| *RIB1*_RV_ deletioncheck | CTTGTAAGAGACCTGCTGTAT |
| *RIB2*_FW_ deletioncheck | GTACACAGTTGTCATTGATGG |
| *RIB2*_RV_ deletioncheck | ACAACTCTGGCAATATACTCC |
| *RIB3*_FW_ deletioncheck | GAGCATTTCAAGCAGAACAAG |
| *RIB3*_RV_ deletioncheck | TGTCTTGTGATAACACCACC |
| *RIB4*_FW_ deletioncheck | TGGTATTATCCATGCTCGTTG |
| *RIB4*_RV_ deletioncheck | CTGCAGGTTCATGATAGCTT |
| *RIB5*_FW_ deletioncheck | GAGTACAACCCTTACGATGAC |
| *RIB5*_RV_ deletioncheck | GCTCTGCAAAGTGACTTCTA |
| *RIB7*_FW_ deletioncheck | CCGTTTGTCACACTGACATA |
| *RIB7*_RV_ deletioncheck | AATTTGACCTCATCTGGTGG |
| **Generation of *C. glabrata* reintegrant strain** | |
| FW *cgRIB1* reintegrant pYC44 | AACCGGATCATCCTACAATTATGTCCCTTCCAGTTGTG |
| RV *cgRIB1* reintegrant pYC44 | TGTCAGTACTGGTCGAGTTATCATATTGTTTCTTGCTCTTG |
| **Generation of *S. cerevisiae* deletion strains** | |
| FW Upstream *RIB1*_KOA1 | AATTACCGCTAAATATGCCTGTCAATCGGATGAAGGGAAGTTTCAACCTCAAATGTAAATAAATAGATGGTGGTCGGCTGGAGATCGG |
| RV downstream *RIB1*_KOA2 | AGAAATAGTAGGGTCTATACCGTTTTTGAAAACAAGTTGTGTAGGGTGAGACTTTTTCTTTTTCCTTTGAGCCGTTATGGCGGGCATC |
| FW Upstream *RIB2*_KOA1 | CAAAATTTTCGGTGGCGCCTTTTTCCGTTTCCAGTAATGAGTGGTTATTGTAGATATGATGTAAGATTAGTGGTCGGCTGGAGATCGG |
| RV downstream *RIB2*_KOA2 | CAGATAACGTCAGTTGTACCACTATCTTTGGTTTTATTCTTTTTATTTTCTCTTCCATATATCTTTACAAGCCGTTATGGCGGGCATC |
| FW Upstream *RIB3*_KOA1 | AGAAAAAGGTCAAAAAACAAAAGATATATACAAAGTTACAAATATATACATGTATATATGTGTGAGTCTGGTGGTCGGCTGGAGATCGG |
| RV downstream *RIB3*_KOA2 | GCTATTGTGAAATTTTCATTGGCGTATATATGTGAGGTGAATGTAAATATCTCCAACAAAAAGCAGAAGGAGCCGTTATGGCGGGCATC |
| FW Upstream *RIB4*_KOA1 | AACAATCAAAGAGCGGGCTTCTTCTATAGTGAGTATATATACATATATAGTATAGATTTCTCTGCGCTTAGTGGTCGGCTGGAGATCGG |
| RV downstream *RIB4*_KOA2 | AAAGTAGCCATATAAAAGAGAACAATGAAATATTGCCCCAGAGAGTGGGTTCAAAAGCATGAAAAGGAACAGCCGTTATGGCGGGCATC |
| FW Upstream *RIB5*_KOA1 | AGTACCAAGTTCTAATCTCATCATTATCACACATTTACAAGACTATTTTTGCAGCAGATGGAGAGATCACGTGGTCGGCTGGAGATCGG |
| RV downstream *RIB5*_KOA2 | TAAAACTAAAGTAGCCGCCCAAGTACTCTCCGGGTACCAATTCCGTCCTTTTCTTTTTTTTCGGTAATTTAGCCGTTATGGCGGGCATC |
| FW Upstream *RIB7* A1 | GAACCATTACATCTGGCTTTGGATACGATGAAACAATCTAGCAGAAATACAGTACCAACCTATTGGTAGCGTGGTCGGCTGGAGATCGG |
| RV downstream *RIB7*_KOA2 | AATAATCTGCAATAAACCAATCACTAGTATGAAAATTTGAAAGGCGCGTTTTTTTCACTAATTTCAGAAAAGCCGTTATGGCGGGCATC |
| FW Upstream *MCH5*_KOA1 | TAAACTGCTTGATGTACGTTTGGAAATTCTCAAATAGCAATTCTCTCTTCAAAGTTAGTGAAGGATAGGTGTGGTCGGCTGGAGATCGG |
| RV downstream *MCH5*_KOA2 | CCCATGATGGTGGCGTAGACATAAAGGGAAGCTGGCCAGTAGATGATATTTATTGGAAAATTTCATCGTTAGCCGTTATGGCGGGCATC |
| *RIB1_* FW_ deletioncheck | AGATATGGGAATTGGCCACA |
| *RIB1_* RV_ deletioncheck | GACTCGTCCTTGAATACGGT |
| *RIB2*_ FW_ deletioncheck | GACGCAGTCATCGGGTACTG |
| *RIB2*_ RV_ deletioncheck | CAGGATCAGTGCCAGCTGG |
| *RIB3*_ FW_ deletioncheck | ACCTTGACGGGCAAGATCAT |
| RIB3_ RV_ deletioncheck | GTTCGATAGTGATTGTTGGGCC |
| *RIB4*_ FW_ deletioncheck | CCAGCATCGCGTCCTGTAGT |
| *RIB4*_ RV_ deletioncheck | CGAACTGGGATGAGGAGTTCTG |
| *RIB5*_ FW_ deletioncheck | CCGCCATCAGTTGGCAGTAG |
| *RIB5*_ RV_ deletioncheck | CGTTACCACCCATAGTAGCAGC |
| *RIB7*_ FW_ deletioncheck | GCGACAACAGTGATTCGAGC |
| *RIB7*_ RV_ deletioncheck | ACTCACCACGAATTGGTTCC |
| *MCH5*_ FW_ deletioncheck | GAATAAACACACATAAACAAACAAAATGAGCTCAGACAGTTTAAC |
| *MCH5*_RV_ deletioncheck | AAATCATAAATCATAAGAAATTCGCTTAAAATCTGACCCACTTGA |

1. Gillum AM, Tsay EY, Kirsch DR. 1984. Isolation of the Candida albicans gene for orotidine-5'-phosphate decarboxylase by complementation of S. cerevisiae ura3 and E. coli pyrF mutations. Mol Gen Genet 198:179-82.

2. Demuyser L, Palmans I, Vandecruys P, Van Dijck P. 2020. Molecular Elucidation of Riboflavin Production and Regulation in *Candida albicans*, toward a Novel Antifungal Drug Target. mSphere 5.

3. Dujon B, Sherman D, Fischer G, Durrens P, Casaregola S, Lafontaine I, De Montigny J, Marck C, Neuveglise C, Talla E, Goffard N, Frangeul L, Aigle M, Anthouard V, Babour A, Barbe V, Barnay S, Blanchin S, Beckerich JM, Beyne E, Bleykasten C, Boisrame A, Boyer J, Cattolico L, Confanioleri F, De Daruvar A, Despons L, Fabre E, Fairhead C, Ferry-Dumazet H, Groppi A, Hantraye F, Hennequin C, Jauniaux N, Joyet P, Kachouri R, Kerrest A, Koszul R, Lemaire M, Lesur I, Ma L, Muller H, Nicaud JM, Nikolski M, Oztas S, Ozier-Kalogeropoulos O, Pellenz S, Potier S, Richard GF, Straub ML, et al. 2004. Genome evolution in yeasts. Nature 430:35-44.

4. Mortimer RK, Johnston JR. 1986. Genealogy of principal strains of the yeast genetic stock center. Genetics 113:35-43.

5. Nguyen N, Quail MMF, Hernday AD. 2017. An Efficient, Rapid, and Recyclable System for CRISPR-Mediated Genome Editing in Candida albicans. mSphere 2.

6. Reuß O, Vik A, Kolter R, Morschhäuser J. 2004. The *SAT1* flipper, an optimized tool for gene disruption in *Candida albicans*. Gene 341:119-27.

7. Demuyser L, Swinnen E, Fiori A, Herrera-Malaver B, Vestrepen K, Van Dijck P. 2017. Mitochondrial Cochaperone Mge1 Is Involved in Regulating Susceptibility to Fluconazole in Saccharomyces cerevisiae and Candida Species. mBio 8.

8. Yanez-Carrillo P, Orta-Zavalza E, Gutierrez-Escobedo G, Patron-Soberano A, De Las Penas A, Castano I. 2015. Expression vectors for C-terminal fusions with fluorescent proteins and epitope tags in Candida glabrata. Fungal Genet Biol 80:43-52.

9. Van Ende M, Timmermans B, Vanreppelen G, Siscar-Lewin S, Fischer D, Wijnants S, Romero CL, Yazdani S, Rogiers O, Demuyser L, Van Zeebroeck G, Cen Y, Kuchler K, Brunke S, Van Dijck P. 2021. The involvement of the Candida glabrata trehalase enzymes in stress resistance and gut colonization. Virulence 12:329-345.

10. Nicolai T, Deparis Q, Foulquie-Moreno MR, Thevelein JM. 2021. In-situ muconic acid extraction reveals sugar consumption bottleneck in a xylose-utilizing Saccharomyces cerevisiae strain. Microb Cell Fact 20:114.

**Supplementary Figure 1**: (A) The growth of *RIB* reintegrant strains was monitored on a medium without riboflavin supplementation. (B) Growth of *RIB* deletion strains on LoFlo medium with 2% glycerol as a carbon source instead of glucose. (C) Growth of reintegrant strains on LoFlo with glycerol. (D) Growth of *RIB* deletion strains on LoFlo with glycerol and 200 mg/L riboflavin. (E) Growth of *RIB* deletion strains on YPD (F) Growth of *RIB* deletion strains on YPD with 200 mg/L riboflavin. The graphs show the mean ± s.d. of three biological replicates.

**Supplementary Figure 2: Riboflavin requirements in an anaerobic condition.** (A) The Wild-strain and *RIB*1 deletions strain were grown in LoFLo medium with varying concentrations of riboflavin supplementation without oxygen present. (B) Growth of a *RIB1* deletion strain in the absence of supplemented riboflavin. To assess cell survival, CFUs were counted every 24 hours on YPD agar plates with 200 mg/L riboflavin to facilitate the growth of the surviving auxotrophic strains.

**Supplementary Figure 3: Fungal burden was assessed after three and six days post-infection.** Mice were intravenously challenged with 8.5 × 10^5^ *C. albicans* cells. (A) Fungal burden in the liver (B) Fungal burden in the spleen.

**Supplementary Figure 4**: (A) Growth of *ScMCH5* orthologous gene deletion strains in the auxotrophic *rib1△/△* background in LoFlo with 100 mg/L of riboflavin compared to auxotrophic *rib1△/△*. Panels B, C, and D show the growth of the wild-type strain SC5314, *rib1△/△,* a sextuple deletion strain in which the genes of five *ScMCH5* orthologs were deleted in the auxotrophic *rib1△/△* background, and five strains similar to the latter strain but with one of the *ScMCH5* orthologous genes remaining in the genome. These strains were grown in 50 mg/L (A), 100 mg/L (B), and 200 mg/L (C) of supplemented riboflavin. The figures show the mean ± s.d. of three biological replicates.

**Supplementary Figure 5**: Overview figure of the relative gene expression of five open reading frames of *ScMCH5* orthologs in different riboflavin concentrations of 0, 20, 50, 100, and 200 mg/L in the wild-type strain and the auxotrophic *rib1△/△* strain. The figures show the mean ± s.d. of three biological replicates, which represent the mean of three technical repeats.


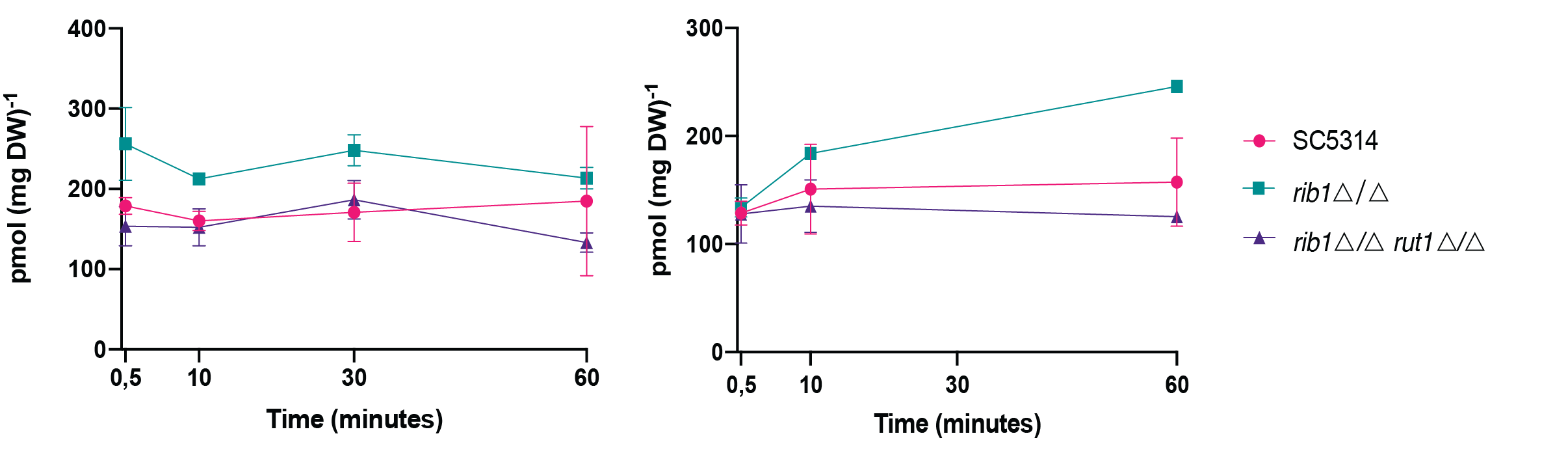


**Supplementary Figure 6**: The uptake of riboflavin in the wild-type, auxotrophic *rib1△/△* and *rib1△/△ rut1△/△* strains is shown in pmol/(mg dry weight) and displayed in time. The two graphs show each a biological repeat with the mean ± s.d. of at least two technical repeats. The experiments were performed on different days.

**Supplementary Figure 7: AlphaFold structure prediction of Rut1.** Dark blue represents a per residue model confidence score (pLDDT) higher than 90, light blue between 70 and 90, yellow between 50 and 70, and orange represents a pLDDT score below 50. According to DeepTMHMM predictions, both the N- and C-termini of Rut1 are located intracellularly.


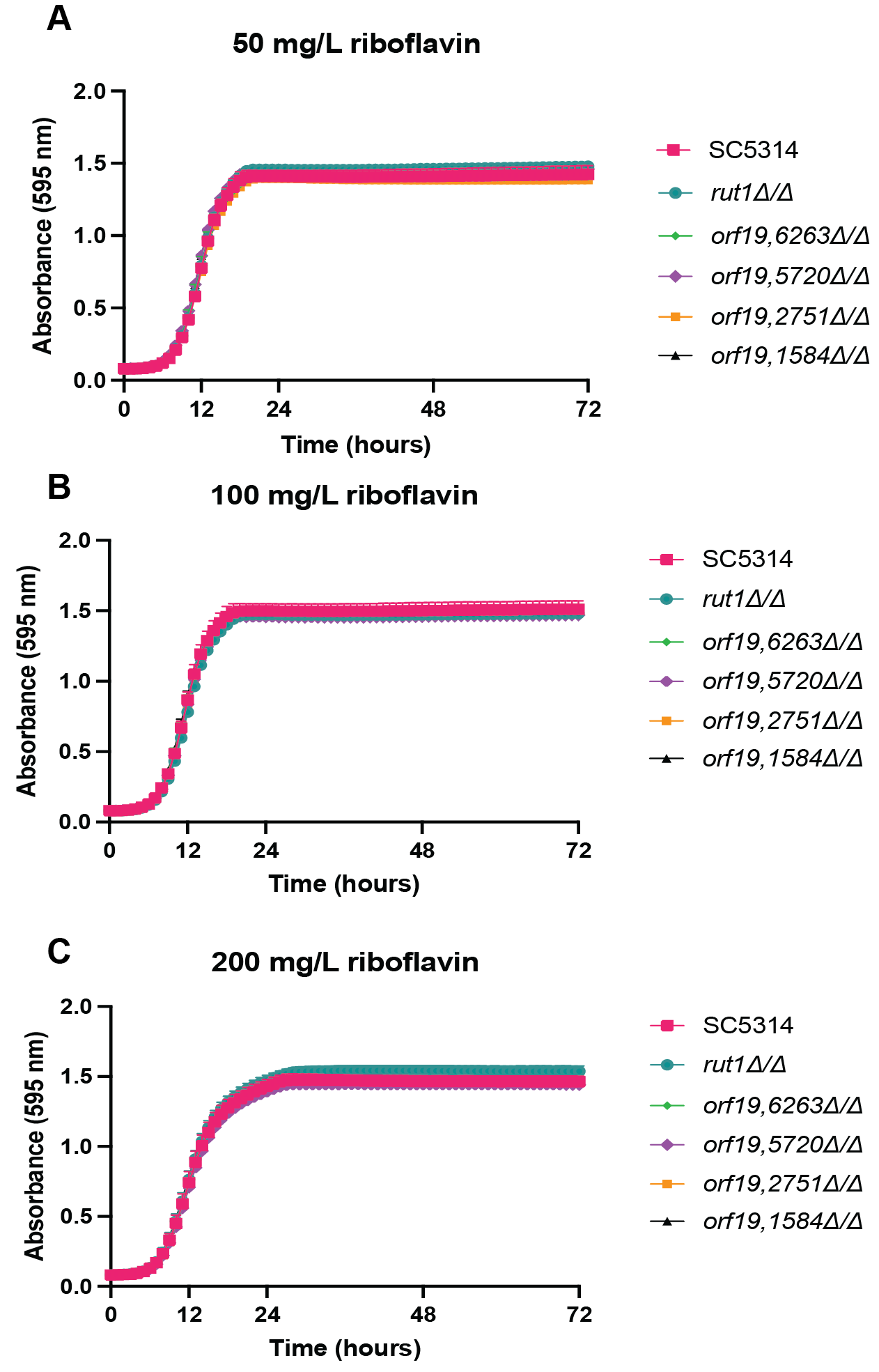


**Supplementary Figure 8**: The growth of single transporter deletion strains in different concentrations of riboflavin: (A) 50 mg/L, (B) 100 mg/L, (C) 200 mg/L.

**Supplementary figure 9: The *RIB1* reintegrant strain behaves simulary to the wild-type strain.** The growth of the wild-type strain, *RIB1* deletion strain and reintegrant strain were monitored for 72h. (A) Growth without riboflavin supplementation (B) Growth in 20 mg/L of riboflavin (C) Growth in 200 mg/L of riboflavin.

**Supplementary Figure 10: Fungal burden of immunosuppressed mice challenged with 5*10^7^ *C.* *glabrata* cells injected via the lateral tail vein.** Fungal burden was assessed two and three post-infection. An additional time point at 12 days post-infection was taken for the *RIB1* deletion strain as they survived the whole experiment. (A) Fungal burden in the liver (B) Fungal burden in the spleen. Statistical analysis was performed using Two-way ANOVA with Tukey's multiple comparisons test *, P < 0.05; **, P < 0.01; ***, P < 0.001; ****, P < 0.0001.
